# Prevalence, pattern and determinants of contraceptive use among pregnant women attending antenatal clinic (ANC) in a secondary health facility in Kebbi State: a cross-sectional study

**DOI:** 10.11604/pamj.2022.41.179.30352

**Published:** 2022-03-07

**Authors:** Ayotunde Sherif Azees, Emmanuel Chukwudi Ehiem, Abdulfattah Isa, Kehinde Joseph Awosan, Aisha Mera Suleiman

**Affiliations:** 1 Department of Community Medicine and Primary Care, Federal Medical Centre Abeokuta, Ogun State, Nigeria,; 2 General Hospital Argungu, Kebbi State, Nigeria,; 3 Department of Epidemiology and Community Medicine, University of Ilorin Teaching Hospital, Kwara State, Nigeria,; 4 Usmanu Danfodio University, Sokoto, Sokoto State, Nigeria

**Keywords:** Contraception, awareness, prevalence, determinants

## Abstract

**Introduction:**

rapidly rising population in Africa is of great concern, especially in Nigeria because of its impact on social stability. Nigeria has been unable to meet the set targets in respect of scaling up access to contraception, and increasing contraceptive prevalence. Thus, being projected to be the third most populous country by 2050 amidst a rising unemployment rate and a shrinking economy.

**Methods:**

a cross-sectional study was conducted among 350 pregnant women attending antenatal clinic (ANC) in General Hospital Argungu, Kebbi State, and data were obtained using an interviewer-administered questionnaire and analyzed using IBM SPSS version 22.

**Results:**

most 318 (90.9%) of the 350 respondents were aware of contraception. The prevalence of previous use of contraceptives among the respondents was 59.4%, while 70% of them intend to use contraception in the future. Concerns about the return of fertility 56 (50.9) after contraceptive use was a major reason given by respondents who had never used any form of contraceptive. Educational attainment, age at marriage, and occupation were the factors that were associated with awareness, previous use, and future use of contraception.

**Conclusion:**

this study underscores the need to promote girl-child education as a cardinal strategy in increasing the level of contraception among the populace.

## Introduction

Africa has the fastest-growing population in the world and is expected to have over 1.8 billion people by 2035 [1]. This ought to be a crucial resource for the continent´s development since it provides resources for the production and consumption of goods and services. However, uncontrolled population growth largely results in a rise in demand for food, water, energy social amenities, and infrastructures necessitating the need for keeping the population at appropriate levels [2]. Family planning (FP) is seen globally as a great public health intervention and its acceptance is rising, however, this is not the case in many African countries [3]. Family planning (FP) has been placed among the cost-effective, and feasible interventions with immediate and long-term benefits for individuals, families, and the nation at large [4]. Studies have identified rising population growth in many African countries as a militating factor to why set developmental goals of eradicating poverty and hunger and reducing maternal and infant mortality are not yet achieved [5]. Family planning protects women from high-risk pregnancies, unwanted pregnancies, unsafe abortions, and sexually transmitted infections including HIV/AIDS through the use of the various contraceptive method available [6-8]. Contraception is not just about limiting family size and spacing of birth, it is about promoting and maintaining the well-being of the mother, child, and that of the family as a whole. Evidence around the world has shown that increased uptake of contraception directly leads to reductions in levels of hunger and poverty. Therefore, it is not surprising that only African countries with high contraceptive prevalence are the ones that have fared better in reducing infant and maternal mortality, and also eliminating hunger and poverty [9]. The current contraceptive prevalence rate among married women in Nigeria is 17%, [10] this is one of the lowest in the continent despite Nigeria´s commitment to attaining a 36% prevalence rate by 2018 from the 15% reported in 2013 [11]. Notwithstanding, the awareness of contraception among women of the reproductive age group in Nigeria remains high despite this low prevalence of contraceptive usage. A previous study in Northwest Nigeria reported an awareness level of 82.4% among women of the reproductive age group, and about half of these women reported current use of contraception [12]. Also, factors that have been found to predict contraceptive use in previous studies include age, education, occupation, marital status, and ethnicity [13,14].

Several institutional and social factors have been reported to influence couples´ decisions on accessing and choices of family planning, these include, non-availability and poor access to contraceptive commodities, non-consenting partners, fear of side effects, and religious considerations. In a previous study in Abia State, 53.2% and 7.9% of the women cited fear of side effects and conflicts with religious belief respectively as reasons why they have never used any contraceptive methods. Contraception does not only affect numbers but prevents unexpected pregnancies and guarantees that all pregnancies are planned, thereby improving quality of life. Also, unplanned pregnancies have been identified as a major contributory factor to maternal deaths in Nigeria [15]. The desire for high numbers of children is traditional to many African cultures and this to date is the practice in several communities in Nigeria, especially in the North. Evidence from the Nigeria Demographic and Health Survey (NDHS) has shown that the desired numbers of children per family in Nigeria range from 3.5 in the Southwest to 5.9 in the Northwest, with Kebbi State having 6.1 which is higher than the average for the region. The areas with high fertility also contribute the most to the maternal and infant mortality figures in the country, with lower figures being reported in areas with lower fertility rates. Therefore, it becomes important to continuously monitor the trend in contraceptive practices in states with high fertility rates and low contraceptive prevalence to determine if progress is being made or otherwise. This will go a long way in tracking progress being made, and as well guide policy-makers and program managers on how to attain future targets on family planning. Therefore, this study aims to determine the awareness of contraception, as well as the prevalence, pattern, and determinants of contraceptive use among pregnant women attending the antenatal clinic (ANC) in General Hospital Argungu Kebbi State, Nigeria.

**Objective:** this study aims to assess the prevalence, pattern and determinants of contraceptive use among pregnant women attending ANC in GH Argungu Kebbi State, Nigeria.

## Methods

**Study design:** a cross-sectional study was conducted among pregnant women attending ANC in General Hospital Argungu, Kebbi State, Nigeria, between June and September 2020. Argungu is one of the four emirates in Kebbi State and it is also the local government headquarter. Argungu is located on latitude: 12° 43' 59.99" N, and longitude: 4° 30' 59.99" E, and has a population of 47,064 people based on the 2006 census, with a projected population of 67,729 people in 2020. Only those who have been pregnant at least once were considered eligible for the study, while those on follow-up visits and those assessed to be requiring urgent medical attention were excluded from the study.

**Sample size:** for the study was calculated using Fisher´s formula for sample size determination for cross-sectional studies [16]. Using a contraceptive prevalence of 26.3% obtained in a previous study in North-Western Nigeria [13], the calculated minimum sample size was 298. To compensate for non-response, an adjustment factor of 15% was used as mark-up [17], bringing the total number of respondents to 350. Antenatal clinics in the facility run twice a week, with Monday being the booking day, and follow-up visits on Wednesday. Only clients who presented in the clinic on Mondays were recruited for the study. The average attendance at each booking clinic in the previous four weeks before the onset of data collection was 74 clients. The sample was spread over 12 weeks, and simple random sampling (using balloting) was employed in selecting respondents on every clinic day till the allotted sample proportion for the day was met. This process was repeated till all the data were collected. A semi-structured interviewer-administered questionnaire was deployed to determine respondents´ sociodemographic characteristics, fertility pattern, awareness, and prevalence of contraceptive use.

The outcome variables were the previous and future contraceptive usage, while the respondents' sociodemographic characteristics were explored as factors that may predict contraceptive usage. Questions were adapted from previously validated tools [10,12,18,19]. The instrument was translated to Hausa and back-translated to English by two independent Hausa scholars. Two midwives in the ANC section of the facility were trained on how to use the instrument, and validation of the instrument was done using the content validity index. The instrument was pretested in 2-selected primary healthcare centers within the local government association (LGA) before actual data collection. Data were collected using the kobocollect app installed on android phones and was sent to the Ona server from where it was uploaded to IBM® SPSS version 22 analysis, all the questions were set as required thereby preventing the risk of having missing data. Quantitative variables were summarized using appropriate measures of central tendency, while categorical variables were summarized using frequencies and proportions. Pearson´s chi-square test and Fisher´s exact were used in assessing for associations between the dependent and independent variables. The significant factors were then subjected to binary logistic regression to control for confounding. All levels of significance were set at p<0.05. Social desirability bias was minimized by explaining to respondents that the study was only for research purposes and not a fault-finding mission, also the various contraceptive methods were stated in terms the respondents clearly understand, preventing misclassification.

**Ethical approval:** ethical approval (FMC/BK/HP/045/P/517) was obtained from the Research and Ethics Committee of Federal Medical Center Birnin Kebbi, and permission was sought from the management of the hospital. Participants in this study were duly informed of the purpose of the study, the benefits, risks, and were assured of confidentiality. Informed consent was obtained from all respondents.

## Results

**Socio-demographic characteristics of respondents:** a total of 350 women who gave consent to participate in the study were interviewed. The mean age of the respondents was 28.8±6.0 years and the majority 224 (64.0%) of the 350 respondents were aged 21 to 30 years, with only nine (2.6%) being above 40 years. The age at marriage ranged from 8 to 37 years (median=18; IQR=5), while two hundred and fifty-eight (73.7%) of the respondents married before 20 years. Almost all the respondents 348(99.4%) were married with only one each that were divorced and widowed. A total of one hundred and eighty (51.4%) of the respondents had formal education, and majority 236 (67.4%) of them were not employed. A hundred and twelve (32%) of the respondents had been married for between 6 and 10 years (Table 1).

**Table 1:** sociodemographic characteristics of respondents (n=350)

Variables	Frequency (%)
**Age group (years)**	
≤ 20	31(8.9)
21-30	224(64)
31-40	86(24.6)
> 40	9(2.6)
**Marital status**	
Married	348(99.4)
Divorced	1(0.3)
Widowed	1(0.3)
**Educational status**	
None	37(10.6)
Quranic	133(38.0)
Primary	43(12.3)
Secondary	117(33.4)
Tertiary	20(5.7)
**Tribe**	
Hausa	303(86.6)
Fulani	13(3.7)
Zabarma	28(8.0)
Yoruba	1(0.3)
Igbo	2(0.6)
Others	3(0.9)
**Occupation**	
Housewife	236(67.4)
Business	95(27.1)
Civil servant	13(3.7)
Others	6(1.7)
**Age at marriage**	
<20	258(73.7)
20-29	91(26.0)
30-39	1(0.3)
**Duration of marriage**	
1-5	68(19.4)
6-10	112(32.0)
11-15	91(26.0)
16-20	50(14.3)
21-25	21(6.0)
>25	8(2.3)

**Respondents´ fertility pattern and awareness of contraception:** a total of two hundred and twenty-seven (64.9%) of the respondents had been pregnant at least 4 times. Most (76.9%) of the respondents had between 1 and 4 children alive, while 81 (23.1%) had at least five children alive. Most 318 (90.9%) of the respondents were aware of contraception, however, nearly all 330 (94.3%) desired to have more than 4 children (Table 2). Assessment of knowledge of benefits of contraception among respondents that were aware of contraception showed that all of them knew that contraceptives help in achieving spacing of childbirth, while only a few of them knew about the other benefits. Among those that were aware of contraception, only 58.2% had sought advice on contraception, and the major source of such advice was at the ANC (78.0%) (Table 2). Respondents age, age at marriage, and educational status were factors that were significantly associated (p<0.05) with being aware of contraception (Table 3).

**Table 2:** respondents' fertility pattern and awareness of contraception

Variables	Frequency (%)
**Numbers of times pregnant(n=350)**	
1-3	123(35.1)
4-6	145(41.4)
7-9	76(21.7)
≥10	6(1.7)
**Numbers of children alive(n=350)**	
1	86(24.6)
2	74(21.1)
3	67(19.1)
4	42(12.0)
≥5	81(23.1)
**Birth interval (months) (n=350)**	
<24	85(24.3)
**≥**24	265(75.7)
**Desired numbers of children(n=350)**	
**1-4**	20(5.7)
**>4**	330(94.3)
**Awared of contraception(n=350)**	
Yes	318(90.9)
No	32(9.1)
**Sources of information about contraceptives (n=318)**	
ANC	273(78.0)
Friends	88(25.1)
Media (radio-tv)	73(20.9)
Spouse	27(7.7)
Family members	61(17.4)
Doctor	11(3.1)
Nurses-midwives	92(26.3)
Others	2(0.6)
**Benefits of contraception(n=318)**	
Spacing of childbirth	318 (100)
Preventing unwanted pregnancy	23 (7.2)
ensuring desired pregnancy	72 (22.6)
Limiting family size	49 (15.4)
**Sought for advice on contraception (n=318)**	
Yes	185(58.2)
No	133 (41.8)
**Places advice were sought (n=185)**	
Anc	146 (78.9)
outpatient clinic	4 (2.2)
family planning clinics	59 (31.9)
patent medicine vendors	6 (3.2)
Others	8 (4.3)
Multiple responses allowed	

**Table 3:** factors associated with awareness of contraception among respondents

Variables	Aware of contraception		Test statistic, p-value
**Age group**	**Yes**	**No**	
≤ 20	23 (74.2)	8 (25.8)	14.635
21-30	206 (92.0)	18 (8.0)	0.006**
31-40	82 (95.3)	4 (4.7)	
> 40	7 (77.8)	2 (22.2)	
**Marital status**			
Married	317 (91.1)	31 (8.9)	10.061
Divorced	0 (0.0)	1 (100)	0.175**
Widowed	1 (100)	0 (0.0)	
**Educational status**			
Informal	139 (81.8)	31 (18.2)	32.898
Formal	179 (99.4)	1 (0.6)	<0.001
**Occupation**			
Housewife	210 (89.0)	26 (11.0)	3.824
Business	89 (93.9)	6 (6.3)	0.259**
Civil servant	13 (100)	0 (0.0)	
Others	6 (100)	0 (0.0)	
**Age at marriage (yrs)**			
<20	229(88.8)	29 (11.2)	5.198
≥20	89(96.7)	3 (3.3)	0.023
** fishers exact			

**Prevalence, pattern and factors associated with contraceptive use among respondents:** the prevalence of previous use of contraceptives among the respondent was 59.4%. Among this, 86.1% were counseled before its use, and about 60% self-decided on the contraceptive method used. The commonest method of contraceptives used were the injectables 115 (55.3%), followed by oral pills 96 (47.6%), and implants 37 (17.8%). None of the respondents had ever used the other methods. Among the respondents that had used contraceptives in the past, most 182 (87.5%) expressed satisfaction with the methods used. Reasons for non-use of any contraceptive method among respondents aware of contraceptives were concern about fertility 56 (50.9%), objection from spouse 19 (17.3%), and fear of side effects 13 (11.8%). The prevalence of contraceptive use before current pregnancy among respondents with a history of previous contraceptive usage was 86.1%. The reasons for not using any method before current pregnancy among those who had previously used a form of contraceptives were concern about fertility 15 (51.7%), irregular bleeding 3 (10.3%), objection from spouse 2 (6.9%), and objection from parents 1 (3.5%). Majority 245 (70.0%) of the respondents expressed intent to use contraceptives in the future, while another 64 (18.3%) were undecided, and 41 (11.7%) have no intention to access contraception in the future. Nearly all (98.3%) of the respondents believed that the hospital is the best place to access contraceptives (Table 4). Age; age at marriage, educational status, and occupation were the factors significantly associated (p<0.05) with the past usage of contraceptives and intent to use contraceptives in the future (Table 5).

**Table 4:** prevalence and pattern of contraceptive use among respondents

Variables	Frequency (%)
**Used contraceptives (n=350)**	
Yes	208 (59.4)
No	142(40.6)
**Reasons for not using contraception among those aware (n=110)**	
Concern about fertility	56 (50.9)
Objection from spouse	19 (17.3)
Fear of side effects	13 (11.8)
Others	31 (28.2)
**Types used (n=208)**	
Injectable	115 (55.8)
Oral pills	96 (46.2)
Implant	37 (17.8)
Traditional method	1 (0.5)
**Counselled before use (n=208)**	
Yes	179 (86.1)
No	29 (13.9)
**Who decided on the type to use (n=208)**	
Self	125 (60.1)
Health workers	51(24.5)
Spouse	23 (11.1)
Family members	6 (2.9)
Joint decision with spouse	3 (1.4)
**Level of satisfaction (n=208)**	
Satisfactory	182 (87.5)
Unsatisfactory	9 (4.3)
Indifferent	17 (8.2)
**Used contraceptives before current pregnancy (n=208)**	
Yes	179(86.1)
No	29(13.9)
**Reason for non-use before current pregnancy(n=29)**	
Concern about fertility	15(51.7)
Irregular bleeding	3 (10.3)
Objection from spouse	2 (6.9)
Objection from parents	1 (3.5)
Other concerns	9 (31.0)
**Plan to use in future**	
Yes	245(70)
No	41(11.7)
Not sure	64(18.3)
*Multiple responses allowed	

**Table 5:** factors associated with past and future usage of contraceptives among respondents

Variables	Past usage		Intent to use in future	
	Yes	No	Yes	No
**Age group**				
≤ 20	10 (43.5)	13 (56.5)	14 (45.2)	17 (54.8)
21-30	129 (62.6)	77 (37.4)	159 (71.0)	65 (29.0)
31-40	64 (78.0)	18 (22.0)	66 (76.7)	20 (23.3)
> 40	5 (71.4)	2 (28.6)	6 (66.7)	3 (33.3)
**Test statistic, p-value**	11.50, 0.008**		11.12, 0.011	
**Marital status**				
Married	207 (65.3)	110 (34.7)	245 (70.4)	110 (34.7)
Unmarried	1 (100)	0 (0.0)	1 (50.0)	1 (50.0)
**Test statistic, p-value**	0.53, 1.0**		0.34, 0.53	
**Educational status**				
Informal	71 (51.1)	68 (48.9)	88 (51.8)	82 (48.2)
Formal	137 (76.5)	42 (23.5)	157 (87.2)	23 (12.8)
**Test statistic, p-value**	22.41, <0.001		52.34, <0.001	
**Occupation**				
Housewife	127 (60.5)	83 (39.5)	150 (63.6)	86 (36.4)
Business	64 (71.9)	25 (28.1)	77 (81.1)	18(18.9)
Civil servant	12 (92.3)	1 (7.7)	12 (92.3)	1 (7.7)
Others	5 (83.3)	1 (16.7)	6 (100)	0 (0.0)
**Test statistic, p-value**	8.93, 0.025**		15.84, 0.001**	
**Age at marriage (yrs)**				
<20	138(60.3)	91 (39.7)	165(64.0)	93 (36.0)
≥20	70(78.7)	19 (21.3)	80(87.0)	12 (13)
**Test statistic, p-value**	**9.58, 0.002**		**17.09, <0.001**	
** fishers exact				

**Predictors of awareness and contraceptive use among respondents:** having formal education was a predictor for being aware of contraception, as respondents with formal education were about 33 times more likely to be being aware of contraception (aOR=32.486, 95%CI= 4.317-244.420, p=0.001). Similarly, respondents with formal education were 3 times more likely to have previously used contraceptives as compared to those without formal education (aOR=2.524, 95%CI=1.530-4.164, p < 0.001). Also, educational attainment, age at marriage, and occupation were predictors of future use of contraceptives. Respondents with formal education were 5 times more likely to use contraceptives in the future, those employed were twice more likely to use contraceptives in the future, while those married at least 20 years of age were also more likely to use contraceptives in the future (Table 6).

**Table 6:** predictors of awareness and contraceptive use among respondents

Variables	AOR	95% Confidence interval		p-value
		Lower	Upper	
**Predictor of awareness of contraception**				
Age (≤ 20 yrs* vs >20yrs.)	2.326	0.898	6.027	0.082
Education (informal* vs formal)	32.485	4.317	244.420	0.001
Age at marriage (< 20 yrs* vs ≥ 20 yrs)	1.647	0.457	5.935	0.445
**Predictor of contraceptive use**				
Education (informal* vs formal)	2.524	1.530	4.164	<0.001
Age at marriage (< 20 yrs* vs ≥ 20 yrs)	1.802	0.986	3.292	0.055
Age (≤ 20 yrs* vs >20 yrs)	1.611	0.654	3.968	0.300
Occupation (Housewife* vs employed)	1.586	0.922	2.730	0.096
**Predictors of intent to use contraceptives in the future**				
Education (informal* vs formal)	4.878	2.812	8.459	<0.001
Age at marriage (< 20 yrs* vs ≥ 20 yrs)	2.433	1.196	4.949	0.014
Age (≤ 20 yrs* vs >20 yrs)	1.482	.656	3.346	0.344
Occupation (Housewife* vs employed)	2.201	1.198	4.047	0.011

## Discussion

This study assessed the awareness, prevalence, pattern, and predictors of contraceptive use among pregnant women attending ANC in a secondary health facility in Kebbi State, Nigeria. A relatively high prevalence of contraceptive use was obtained in this study as compared to what was reported in the 2018 NDHS, [10] this may be because the study was conducted in an urban LGA and shows the disparity that exists in the utilization of family planning services between urban and rural areas [20]. The prevalence of 59.4% reported in this study is similar to that reported in Lagos (57.6%) which is also an urban area, [19] but higher than the 26.3% reported in Birnin Kudu a semi-rural LGA in Jigawa State [13]. Age; age at marriage, educational attainment, and employment status were the factors associated with previous use of contraception among respondents, however, only education attainment was a predictor of previous use of contraception as those with formal education were found to be 32 times more likely to have previously used one form of contraceptives or the other. Similar findings were reported in studies in Jigawa and Oyo States, [13,14] and underscore the relevance of girl child education as a way of reducing the burden associated with unplanned pregnancies. Contrary to findings in Lagos, Aba, and Calabar, all in the southern part of the country, a high proportion (94.3%) of women in this study were desirous of having more than four children. This is not surprising as finding from the 2018 NDHS shows higher fertility rates for states in the north as compared to the south [10]. In similarity to findings in previous studies in Nigeria, [12,21-23] women in this study had a high level of awareness of contraception. The high level of awareness ordinarily should be encouraging as available evidence suggest awareness to be the first point in the continuum leading to contraceptive use, [24,25] however, this was not the case as one would have expected a correspondingly higher level of past contraceptive usage among respondents. The study by Adefalu *et al*. reported a similar finding [12]. The non-concurrence between contraceptive awareness and usage in both studies may be due to gaps in knowledge of contraception and its safety, hence the need for enlightenment campaigns targeted at educating couples on the safety of the various contraceptive methods.

The ante-natal clinic was the main source of information on contraception for most of the respondents in this study, this is similar to finding in another study in Northwest Nigeria, [12] however, this contrast to findings in Anambra State and another in Southwest Nigeria where the mass media was the main source of information on contraception [26,27]. The mass media has become a major source of information dissemination in the 21^st^ century because of its speed, reach, and diversity and hence can be very useful in disseminating information on contraception, therefore program planners need to take advantage of this opportunity in reaching the target population when developing programs aimed at improving contraceptive uptake. The poor knowledge of the benefits of contraception among the respondents in this study despite a high awareness suggests gaps in the contents of the health talks during antenatal clinics, as this was the main source of information for most of the respondents. Interestingly, a good number of the women had at one point in time sought more information on contraception showing their desire to know more about contraception, this contrasts with findings in Abia State where it was reported that fewer women sought more information on contraception [18]. This further underscores the role ANC plays in disseminating information on contraception and service providers can leverage this to improve clients' knowledge on contraception. Although the respondent's age, age at marriage, and educational attainment were associated with awareness, only their education status predicts awareness of contraception, with respondents with formal education being about 33 times more likely to be aware of contraception when compared to those with informal education. Other studies in Nigeria have reported a similar association, [12,21,28] and this underscores the importance of girl child education as a strategy for increasing contraceptive awareness among the populace.

Allowing clients to make informed choices on the method of contraception used is a way of promoting clients' satisfaction, and such decisions should be jointly made with their spouses [29]. Contrary to findings in the 2018 NDHS where two-thirds of the respondents were said to have decided jointly with their spouses, [10] almost two-thirds of the women in this study self-decided on the choice of contraceptive methods used. This implies low male involvement in the decision-making process regarding the choice of contraceptive methods used by women in this study which may lead to high discontinuation rates among users if the spouse is dissatisfied with the chosen method. Hence, the need for better male involvement. In similarity to the finding in Birnin Kudu, Jigawa State, [13] injectables were the commonest form of contraceptives used by respondents in this study. Although this may not be ideal, it is not surprising because evidence has shown uptake of the long-acting contraceptive methods to be generally low in Nigeria due to poor knowledge on its use and safety profile [30]. Concerns about fertility, objection from spouses, fear of side effects, and irregular bleeding were the major reasons why some respondents in this study were not using contraception, these were not different from reports in previous studies [18,28]. This further underscores the need for educating the populace on the safety of contraceptive methods and promoting male involvement as a mainstay in increasing contraceptive prevalence. Another significant finding in this study is that seven out of every ten respondents intend to use contraceptives in the future; this is similar to the finding in a study in Kaduna state, which is also in Northern Nigeria [28]. This is encouraging and it provides additional evidence that the populace is beginning to appreciate the benefit of contraceptives and this is expected to increase as their knowledge increases. Also, the fact that educational attainment was a major factor that predicts intent to use contraceptives in the future among women in this study further underscores the pivotal role education plays in contraceptive usage.

## Conclusion

The prevalence of past contraceptive use among respondents was relatively high, and the majority of the respondent expressed intent to use contraceptives in the future. Educational attainment was a major determinant for both past and future contraceptive use. However, despite the relatively high past adoption of contraception, nearly all the women were desirous of having more than four children. Concerns about fertility, spousal objection, and fear of side effects were the major reasons expressed by women who had not used any form of contraception in the past. Therefore, promoting girl child education and male involvement in contraception as well as using diverse means in communicating the benefits of contraception will go a long way in improving contraceptive prevalence and acceptability.

### What is known about this topic

- *Awareness of contraception is high among women of the reproductive age group;*
- *Contraceptive prevalence is low in Nigeria, with states in the northwest reporting the lowest prevalence rates*.

### What this study adds

- *The study showed that contraceptive prevalence is increasing gradually;*
- *The study shows that male involvement in contraception is still poor;*
- *The study shows the role of education in increasing contraceptive prevalence*.
